# A Benzimidazole Proton Pump Inhibitor Increases Growth and Tolerance to Salt Stress in Tomato

**DOI:** 10.3389/fpls.2017.01220

**Published:** 2017-07-18

**Authors:** Michael J. Van Oosten, Silvia Silletti, Gianpiero Guida, Valerio Cirillo, Emilio Di Stasio, Petronia Carillo, Pasqualina Woodrow, Albino Maggio, Giampaolo Raimondi

**Affiliations:** ^1^Department of Agricultural Sciences, University of Naples Federico II Naples, Italy; ^2^National Research Council of Italy, Institute for Agricultural and Forestry Systems in the Mediterranean (CNR-ISAFoM) Ercolano, Italy; ^3^Department of Environmental, Biological and Pharmaceutical Sciences and Technologies, University of Campania “Luigi Vanvitelli” Caserta, Italy

**Keywords:** benzimidazole, chemical priming, omeprazole, proton pump inhibitor (PPI), salt stress

## Abstract

Pre-treatment of tomato plants with micromolar concentrations of omeprazole (OP), a benzimidazole proton pump inhibitor in mammalian systems, improves plant growth in terms of fresh weight of shoot and roots by 49 and 55% and dry weight by 54 and 105% under salt stress conditions (200 mM NaCl), respectively. Assessment of gas exchange, ion distribution, and gene expression profile in different organs strongly indicates that OP interferes with key components of the stress adaptation machinery, including hormonal control of root development (improving length and branching), protection of the photosynthetic system (improving quantum yield of photosystem II) and regulation of ion homeostasis (improving the K^+^:Na^+^ ratio in leaves and roots). To our knowledge OP is one of the few known molecules that at micromolar concentrations manifests a dual function as growth enhancer and salt stress protectant. Therefore, OP can be used as new inducer of stress tolerance to better understand molecular and physiological stress adaptation paths in plants and to design new products to improve crop performance under suboptimal growth conditions.

**Highlight:** Omeprazole enhances growth of tomato and increases tolerance to salinity stress through alterations of gene expression and ion uptake and transport.

## Introduction

Soil salinization is a major problem for agriculture. It is estimated that by 2050 salinization will lead to up to 30% degradation of cultivated land (Rengasamy, 2006; FAO, 2011; Aragüés et al., 2015; Lal, 2015). The effects of soil and water salinity on plant growth and development have been well-documented. Excess of Na^+^ and Cl^-^ ions in proximity of the roots generate osmotic and ionic stress and activate signals inhibiting cell division and plant growth (Deinlein et al., 2014). Metabolic dysfunction and nutritional disorders associated with Na^+^ and Cl^-^ loading in plant tissues and organs translate in further growth reduction and eventually irreversible cell damage. Upon exposure to salt stress, the control of growth, ion and water homeostasis becomes an essential part of an adaptation program that helps resuming growth, albeit at a reduced rate (Maggio et al., 2006; Park et al., 2016; Van Oosten et al., 2016; Annunziata et al., 2017). During adaptation, ion movement through cellular compartments is essential to detoxify the cytoplasm and re-establish osmotic balance (Hasegawa, 2013; Ji et al., 2013; Mancarella et al., 2016). Plasma membrane and vacuolar H^+^-ATPases play a fundamental role in this physiological process since by generating active transport of proton H^+^ across the membranes they create pH gradients and electrical potentials that drive transport of ions and molecules (including NO_3_^-^, PO_4_^-^, K^+^, Na^+^, sucrose, hexoses, and amino acids) across membranes (Pardo et al., 2006; Batelli et al., 2007; Deinlein et al., 2014). H^+^-ATPases can be activated/deactivated in response to many environmental cues such as abiotic stresses (Hasegawa, 2013). It occurs that salinization stimulates vacuolar H^+^-ATPase and H^+^-PPase activities which in turn facilitate tonoplast Na^+^/H^+^ antiporter function and cytoplasm detoxification (Pardo et al., 2006; Ji et al., 2013). It has been shown that increasing cellular proton pump activity via co-overexpression of the vacuolar H^+^-pyrophosphatase gene AVP1 and the vacuolar Na^+^/H^+^ antiporter gene AtNHX1, enhanced salt stress tolerance, most likely by potentiating ion compartmentalization functions (Shen et al., 2015). In contrast, cell treatment with vanadate (an inhibitor of the plasma membrane H^+^-ATPase) increases the Na^+^/K^+^ ratio in plant tissues and enhances sensitivity to salinity (Li et al., 2014).

In animals, homologs of plant proton pumps operate through the H^+^/K^+^ ATPase mechanism (Axelsen and Palmgren, 1998). Similar to plants, animal proton pumps working across membranes generate acidification of organismal compartments. For decades proton pump inhibitors (PPIs) have been successfully used to inhibit gastric acid secretion (McTavish et al., 1991). As matter of fact, benzimidazole based PPIs are common treatments used with gastro-esophageal reflux disease (GERD) and peptic ulcers (Baumann and Baxendale, 2013). In animals, omeprazole, the most common benzimidazole PPI, affects P-Type IIC ATPases. These P-Type IIC ATPases represent a large family of ATP driven transporters, which are responsible for moving ions across membranes. These include membrane Ca^2+^ pumps, Na^+^/K^+^ transporters, and H^+^/K^+^ transporters. Omeprazole is largely used as PPI that suppresses stomach acid secretion in the gastric mucosa (Wallmark, 1986; Shin et al., 2009). The specific inhibition of the P-Type IIC H^+^/K^+^ ATPase located in the parietal cells is irreversible and specific. Plants are not known to possess P-Type IIC ATPases that transport Na^+^ or K^+^, instead relying on the family of NHX-type Na^+^ and K^+^/H^+^ antiporters for plasma membrane extrusion and compartmentation into the vacuoles and endosomes. Plants do possess P-Type IIA and III ATPases, primarily SERCA-like, which are not known to transport Na^+^ or K^+^. SERCA-like ATPases show very low homology (approximately 25%) to P-Type IIC ATPases that transport Na^+^ or K^+^ (Sweadner and Donnet, 2001). The SERCA-like ATPases are primarily endoplasmic reticulum transporters of calcium (Altshuler et al., 2012) and manganese (Mills et al., 2008). Plants also have P-Type III ATPases typically found in the plasma membrane, but none have been functionally characterized as Na^+^ or K^+^ transporters. While plants are not known to possess P-Type IIC ATPases that transport Na^+^ or K^+^ which are the target of omeprazole, we wanted to verify whether plant treatment with omeprazole may actually alter the transmembrane control of ion fluxes and disrupt plant tolerance to saline stress. In contrast to what we may have expected based on our current understanding of plant ATPases and plant responses to salt stress, here we demonstrate that tomato treatment with micromolar concentrations of omeprazole greatly enhanced plant growth and improved its tolerance to saline stress.

## Materials and Methods

### Plant Growth Conditions

#### Hydroponic Experiment

Tomato seeds (cultivar M82, accession LA3475) obtained from the Tomato Genetics Resource Center (TGRC^1^) were germinated in plates containing MS media and transplanted to a hydroponic system after 1 week when cotyledons were fully expanded. Air temperature (T_a_, °C), humidity (RH, %), and solar radiation (R_s_, W m^-2^) were acquired by a data logger (Spectrum Technologies, Plainfield, IL, United States). The average air humidity and temperature were 58% and 24°C during the day and 86% and 18°C during night-time, under short day conditions. Plants were grown in hydroponic solution containing: 1.5 mM Mg(NO_3_)_2_ ∙ 6H_2_O, 3.4 mM Ca(NO_3_)_2_ ∙ 4H_2_O, 1 mM KNO_3_, 1.8 mM K_2_SO_4_, 1.5 mM KH_2_PO_4_, and 14 mg/L Hidromix [Valagro, Atessa (Chieti) Italy]. Eight plants per treatment were grown in 4 L tanks with constant aeration. At 26 Days After Sowing (DAS), the first treatment of OP was added to the nutrient solution (1, 10, and 45 μM). Salt stress was initiated 36 DAS by adding 75 mM NaCl to salt treatments. At 42 DAS the NaCl concentration was increased to 150 mM and on 46 DAS the NaCl concentration was raised to 200 mM NaCl. Destructive harvest and biometrics were taken at 50 DAS (14 days of salt stress). Roots were separated from shoots in order to obtain their individual FW. To measure relative water content (RWC), shoots were transferred in deionized water for 24 h to induce maximum turgidity and weighed. Shoots and roots were dried for 3 days at 64°C and weighed individually. Root length was measured with a ruler; root area was measured using ImageJ as per Yoo et al. (2010).

#### Soil Experiment

Seedlings were germinated in previously stated growth conditions and when seedlings had four true leaves, they were transplanted into 5 L pots, filled with soil and fertilized after 7 days with Nitrophoska gold (Compo Agricoltura, Cesano Maderno, Italy). Then plants were equally divided into control and stress treatments, six replicates per treatment, and arranged in a randomized block design. Plants were well-irrigated for 30 days prior to start of the stress treatments. OP treated plants were watered with 500 mL of 1 μM omeprazole at 36, 50, and 68 DAS. At 60 DAS pre-stress physiological measurements were taken and then pots were saturated with 50 mM NaCl and the final salt concentration of 150 mM was achieved at 68 DAS. At 72 DAS (14 days of salt stress), photos and physiological measurements were carried out.

### RNA Extraction and Quantitative RT-PCR

Leaves of 7-week-old hydroponically grown plants (50 DAS, 14 DAST), treated with 1, 10, and 45 μM OP, with 0 and 200 mM NaCl, were harvested and immediately frozen at -80°C. Leaves from the same treatment were mixed and three replicates per bulk were analyzed. 100 mg of fresh leaf tissue per sample was homogenized with liquid nitrogen and extracted with 1 ml of TRIzol (Life Technologies). First-strand synthesis was performed with a QuantiTect Reverse Transcription Kit (QIAGEN) using 1 μg of total RNA. Real-time qPCR reactions, using 10 ng of cDNA per reaction, two experiments, four replicates per experiment, were carried out on an ABI 7900HT qPCR detection system using Platinum SYBR Green qPCR SuperMix-UDG with ROX (Life Technologies). Each qRT-PCR experiment was repeated at least twice to confirm results. Primers were designed based on gene models and EST sequences available in GenBank^2^. All qRT-PCR primers were determined to be within 3% efficiency of each other. Relative expression levels were calculated using EF1α as an internal standard and the ^ΔΔ^Ct method for relative quantification. The primers used are listed in Supplementary Table S1.

### Ion Measurements

Ions measurements were performed according to a procedure described by Carillo et al. (2011), with following modifications. 100 mg of powdered dried material was suspended in 10 mL of MilliQ grade water (Milli-Q PLUS, Millipore, United States), and subjected to four freeze-thaw cycles by freezing in liquid nitrogen and thawing at 40°C. Samples were centrifuged at 34000 × *g* for 10 min and the clear supernatants were analyzed by ion-exchange chromatography using a DX500 apparatus (Dionex, Olten, Switzerland) with an IONPACATC1 anion trap column (Dionex), an IONPAC-AG11 guard column (Dionex) and an analytical IONPAC-AS11 4-mm column (Dionex), fitted with an ASRSII 4-mm suppressor for anions (Dionex), and an IONPAC-CTC cation trap column (Dionex), an IONPAC-CG12A guard column (Dionex) and an analytical IONPAC-CS12A 4-mm column (Dionex), fitted with a CSRS 4-mm suppressor for cations (Dionex), with detection by a CD20 conductivity detector (Dionex), according to the manufacturer’s instructions.

### Chl *a* Fluorescence Emission and Gas Exchange

To determine chlorophyll *a* (Chl *a*) fluorescence, a LED light source with emission peaks centered at 465 (blue) and 635 nm (red) generated a PPFD equal to 1500 μmol (photons) m^-2^ s^-1^ (90% red, 10% blue). A modulated fluorometer analyzer, Li-6400XT (Li-Cor Biosciences, Lincoln, NE, United States), was used to assess the fluorescence parameters. The measuring beam was set at intensity 5 (according to the instrument manual) with a modulation of 20 kHz. After the measurement of *Chl a* fluorescence emission at steady-state under light conditions, F′, the maximum fluorescence emission, F_m_′, was assessed upon induction by a 0.8 s saturating light pulse at 6000 μmol (photons) m^-2^ s^-1^ at 20 kHz. After that, actinic light was briefly switched off while a far-red light of 8 μmol (photons) m^-2^ s^-1^ for 6 s was used to discharge the PSII to allow measurement of the minimum fluorescence emission under light conditions, F_0_′. Photochemical quenching (qP) was calculated from the previously mentioned parameters. Net photosynthetic CO_2_ assimilation rate (A, μmol m^-2^ s^-1^) and stomatal conductance of water vapor (*g*_s_, mol m^-2^ s^-1^) were measured using a portable open-system gas-exchange (Li-6400XT).

Measurements of photosynthetic rates (A) and stomatal conductance (gs) were taken at saturating light on well-exposed and fully expanded top leaves of six plants per treatment. Leaf chamber CO_2_ was set to 400 μmol CO_2_ mol^-1^ air. Measurements were taken for each time point between 10:00 and 13:00 for the duration of the experiment (November 10th–23rd, 2016). Gas-exchange parameters using the von Caemmerer and von Farquhar (1981) model were calculated by instrument software (Li-Cor, 2011). The effective quantum yield of PSII photochemistry in light**-**adapted leaves was calculated using: Φ_PSII_ = (F_m_′– F′)/F_m_′ (Genty et al., 1990). Anatomical analysis on a leaf area of 0.069 mm^2^ were performed as described in Yoo et al. (2010).

### Statistical Analysis

Biometric measurements were statistically analyzed using the Student’s *t*-test. Photosynthesis and gas exchange results were statistically analyzed using two-way ANOVA procedure with Sidak multiple comparisons test. Ion quantification was analyzed using a two-way ANOVA and Duncan’s multiple range to determine differences between means (*P* ≤ 0.05). Ions for roots and shoots were statically analyzed in two separate analyses.

## Results

### Plant Growth

**FIGURE 1:**
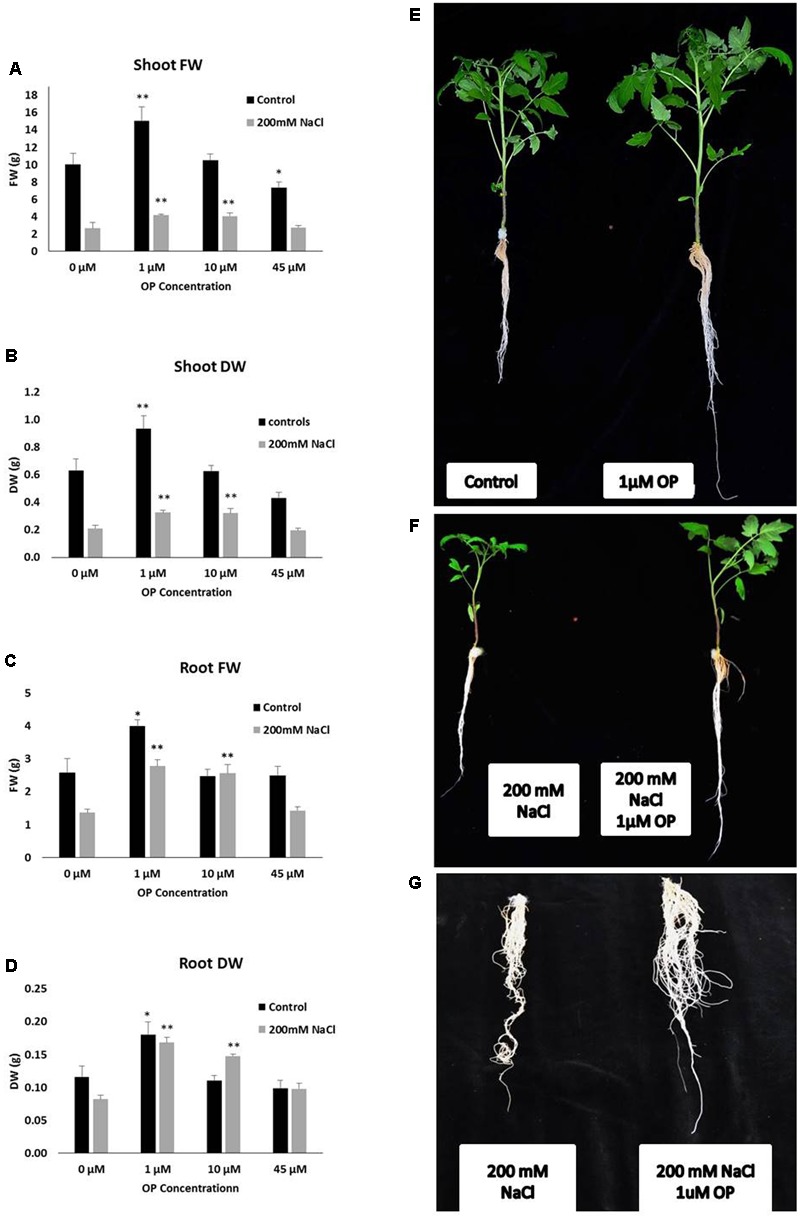
Omeprazole (OP) enhances shoot and root growth of *Solanum lycopersicum* var. Red Setter plants under control and salt stress conditions. Photos (right panel series) show representative plants **(E,F)** or roots **(G)** treated with 1 μM OP and controls, with and without 200 mM NaCl. The left panel series indicates Shoot fresh weight (FW) and dry weight (DW) **(A,B)** and Root FW and DW **(C,D)**. Plants were grown in a hydroponic solution containing 0, 1, 10, and 45 μM OP, with and without 200 mM NaCl. Plants were harvested after 2 weeks of salt treatment (50 DAS, 14 DSS) and average shoot and root FW and DW was calculated. Values indicate average ± SE (*n* = 7). Single asterisks denote significant differences according to Student (*P* < 0.1) between untreated controls and OP treated plants, double asterisks denote (*P* < 0.01) between untreated controls and OP treated plants.

**FIGURE 2:**
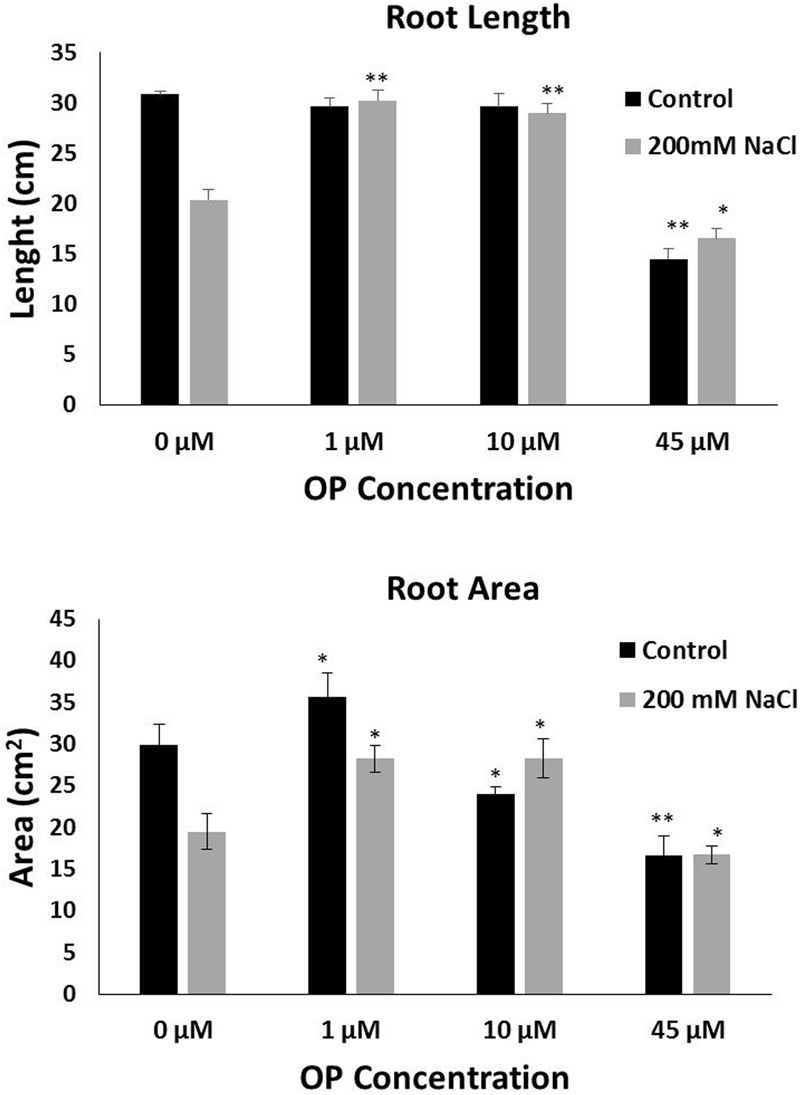
Omeprazole enhances root growth of *Solanum lycopersicum* var. Red Setter plants under control and salt stress conditions. Average Root Length **(top)** and Average Root Area **(bottom)**. Plants were grown in a hydroponic solution containing 0, 1, 10, and 45 μM OP, with and without 200 mM NaCl. Plants were harvested after 2 weeks of salt treatment (50 DAS, 14 DSS) and root length and area were measured. Values indicate average ± SE (*n* = 7). Single asterisks denote significant differences according to Student (*P* < 0.1) between untreated controls and OP treated plants, double asterisks denote (*P* < 0.01) between untreated controls and OP treated plants.

In order to assess the potential for omeprazole to affect growth and salt tolerance, we conducted a hydroponic experiment at increasing OP concentrations. Measurements were taken to separate phenotypes of roots and shoots. Our results indicate that OP induces two specific phenotypes: (1) increased growth of roots and shoots and (2) increased tolerance and growth under high salt stress. Specifically, for growth we observed that OP works in a dose dependent manner (**Figure 1**). Low doses, 1 μM, stimulated significant increases in growth while a higher dosage either had no stimulatory effect (10 μM) or, in the case of 45 μM, were inhibitory to growth. Application of OP at 1 μM resulted in 49 and 48% increases in shoot FW and DW, respectively (**Figure 1**). The highest dose (45 μM) inhibited growth of shoots and reduced FW and DW by 37 and 32%, respectively (**Figure 1**). The stimulatory effect of OP was not limited to shoots; we also observed that 1 μM stimulated root growth and biomass accumulation by increasing FW and DW of roots by 55 and 56%, respectively. Higher concentrations, 10 and 45 μM, did not significantly affect root growth (**Figure 1**). In control growth conditions, low concentrations of OP did not increase root length significantly. However, at 45 μM root length was severely inhibited, with a reduction of 53% (**Figure 2**). While OP did not significantly increase the maximal lengths of roots, low doses greatly increased root mass. OP also had effect on later root branching resulting in changes to the overall root area. Low concentrations of 1 μM increased root area by 19%. Again, 45 μM had an inhibitory effect, decreasing root area by 44% (**Figure 2**).

### Salt Stress Tolerance

The role of OP on salt stress tolerance was assessed in hydroponic culture. OP had significant effects on shoot growth and remarkable effects on root growth in the presence of NaCl stress. Plants grown under severe salt stress, 200 mM NaCl, and low concentrations of OP were able to maintain growth. Treatment with 1 μM under 200 mM NaCl increased shoot FW and DW over untreated controls by 56 and 54%, respectively (**Figure 1**). Similar results in shoots were observed with 10 μM treatments and salt stress. The application of 45 μM did not increase either of these shoot growth parameters under severe salt stress. Growth promotion under severe salt stress was more pronounced in roots. Both root FW and DW of 1 μM OP treated plants was double (103 and 105%, respectively) that of untreated controls under severe salt stress (**Figure 1**). While 10 μM had no significant effect in control conditions, we observed increased tolerance in salt stress conditions. Salt stressed plants treated with 10 μM demonstrated increases of 52% of FW and DW over untreated plants subjected to 200 mM NaCl (**Figure 1**). High concentrations of OP (45 μM) did not induce significant gains under salt stress. Average root length for 1 and 10 μM treated plants under salt stress was similar to controls and unstressed plants, showing no reduction in root length, while salt stress reduced average root length in untreated controls by one third (**Figure 2**). OP treated plants also showed increased root area under severe salt stress, with 1 and 10 μM treated plants having an average of 45% more root area that untreated controls (**Figure 2**). Treatment with OP did not significantly alter RWC of leaves in either control or salt stressed plants (90 ± 0.04% for controls and 88 ± 0.04% for OP treated plants and 68 ± 0.03% for salt stressed plants and 71 ± 0.01% for salt stressed plants treated with OP).

### Gas Exchange and Chl *a* Fluorescence Emission

Gas exchange and Chl *a* fluorescence emission were measured to assess direct effects of OP treatment on these physiological parameters. For this purpose, we conducted a second experiment with plants grown in soil. The second soil experiment was conducted using what was deemed to optimal concentration for OP (1 μM) and a NaCl concentration of 150 mM. Before stress imposition, A, Net photosynthetic CO_2_ assimilation rate (A, μmol m^-2^ s^-1^) and g_s_ stomatal conductance of water vapor (*g*_s_, mol m^-2^ s^-1^) did not show any significant difference with respect to OP treatments, and they averaged 25.8 and 0.397 μmol m^-2^ s^-1^, respectively. In non-stress conditions, we did not observe any effect by OP on gas exchange or photosynthesis. Before salt stress imposition there was no statistical difference between untreated controls and OP treated plants where the average, Φ_PSII_ and *qP* were 0.255 and 0.456 respectively.

**FIGURE 3:**
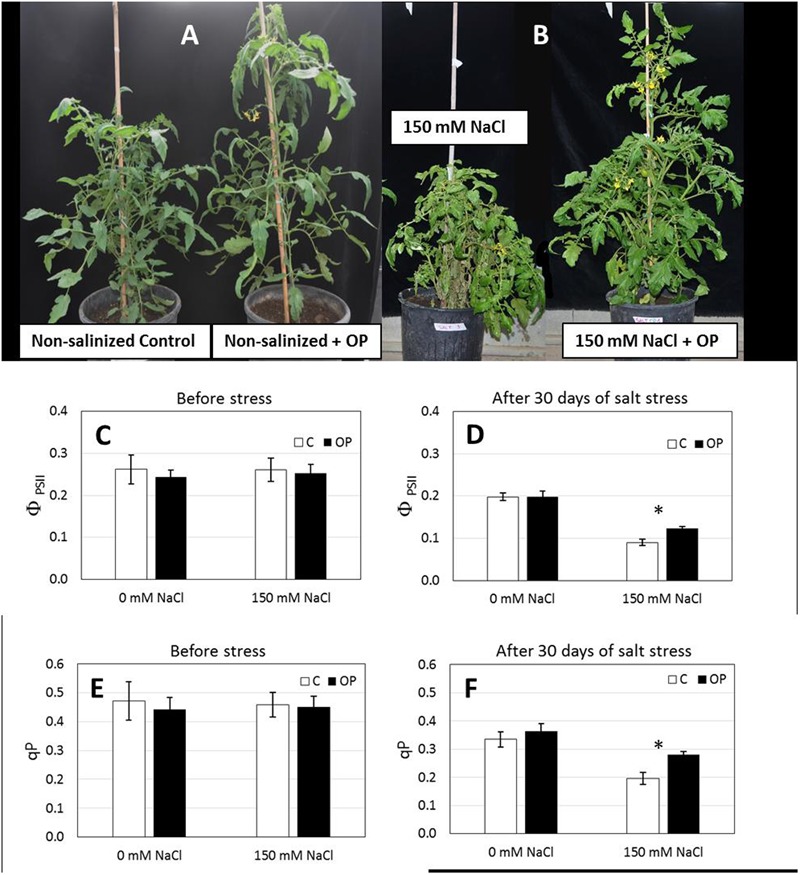
Omeprazole enhances growth and tolerance to salt stress of *Solanum lycopersicum* var. Red Setter plants. Plants were grown in soil, unsalinized or salinized with 150 mM NaCl and irrigated with 0 and 1 μM OP. Photos **(A,B)** of representative plants were taken after 2 weeks of salt treatment (72 DAS, 14 DSS) and efficiency of Photosystem II (Φ_PSII_, **C,D**) and photochemical quenching (qP, **E,F**) was measured. Values indicate average ± SE (*n* = 6). Single asterisks denote significant differences according to Student (*P* < 0.01) between untreated controls and OP treated plants.

After imposition of salt stress with 150 mM NaCl, we observed no significant differences for A and g_s_ under salt stress conditions in soil, between OP treated plants and controls (Supplementary Figure S1). However, we did observe a protective effect on photosystem integrity in 1 μM OP treated plants subjected to salt stress. Both φ_PSII_ (quantum yield of photosystem II) and *qP* (photochemical quenching) were significantly affected by salt stress and OP (**Figure 3**). After 14 days of salt stress Φ_PSII_ was 0.124 and *qP* 0.279 in OP treated plants, 37 and 43%, respectively, higher than untreated controls (**Figure 3**). Stomatal Index and Stomatal Density were found to be similar between OP treated plants and controls (data not shown).

### Ion Profile of Omeprazole Treated and Salt Stressed Plants

**FIGURE 4:**
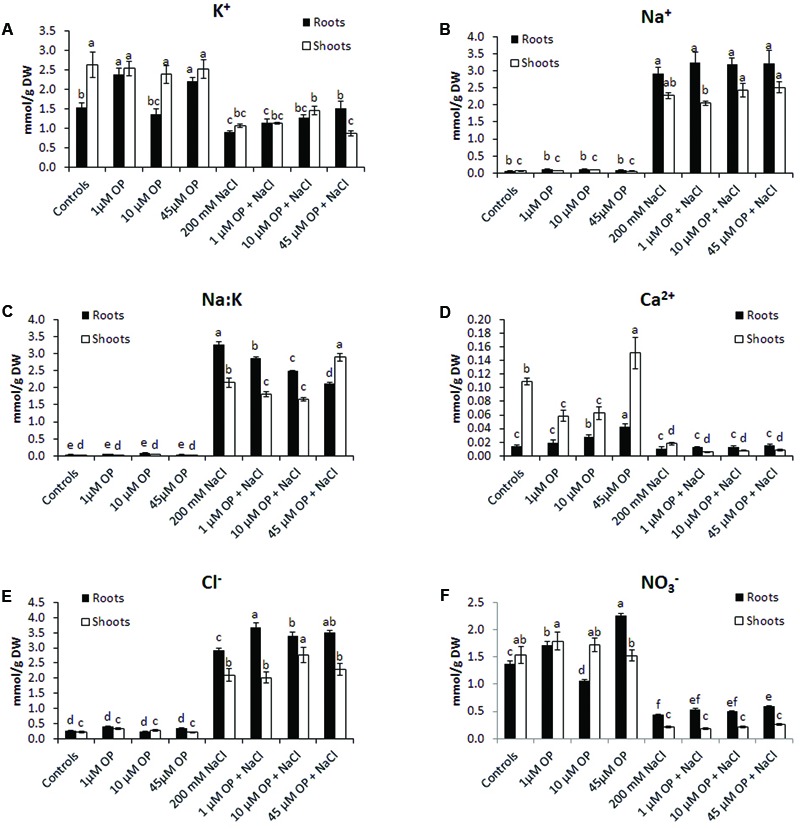
Ion profiles of *Solanum lycopersicum* var. Red Setter plants treated with omeprazole. Plants were grown in a hydroponic solution containing 0, 1, 10, or 45 μM OP, with and without 200 mM NaCl. Plants were harvested after 2 weeks of salt treatment (50 DAS, 14 DSS) and used for ion analysis. Values for K^+^ **(A)**, Na^+^ **(B)**, Na:K ratio **(C)**, Ca^2+^ **(D)**, Cl^-^**(E)**, and NO3^-^**(F)** are shown. Values indicate average ± SE (*n* = 6). Different letters indicate significant differences at *P* < 0.05 between an OP treated sample and the corresponding untreated control.

To better understand the mechanisms that OP affects to increase growth and tolerance to salt stress, tissue ion concentrations were profiled in all hydroponic treatments. OP altered the ion accumulation of tomato plants in control conditions and under severe salt stress. In unstressed conditions, OP increased K^+^ accumulation in roots treated with 1 μM (**Figure 4**); however, it did not increase Na^+^ accumulation. OP did affect the Na^+^:K^+^ ratio of salt stressed leaves and roots. The root Na^+^:K^+^ ratio of roots under salt stress was reduced by 12, 23, and 35% in 1, 10, and 45 μM OP treated plants, respectively (**Figure 4**). Calcium accumulation was also affected by OP treatment. In shoot, lower OP concentrations, 1 and 10 μM, decreased shoot calcium concentration significantly. Interestingly, 45 μM OP increased calcium concentration in roots and shoots. Root chloride accumulation was observed to be elevated in roots of plants treated with OP when compared to controls only under stress condition. Treatment with OP was observed to increase nitrate content of roots at 1 and 45 μM. OP treatment with salt stress did not result in any significant increase in nitrate accumulation (**Figure 4**).

### Gene Expression

**FIGURE 5:**
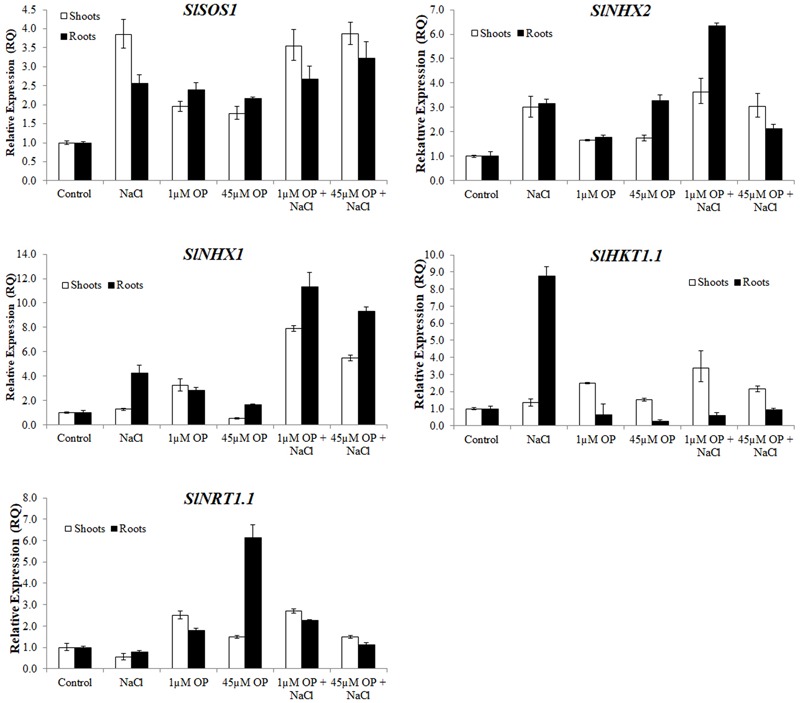
Ion transporter gene expression in plants treated with omeprazole. Plants were grown in a hydroponic solution containing 0, 1, and 45 μM OP, with and without 200 mM NaCl. Samples for qRT-PCR were harvested after 2 weeks of salt treatment (50 DAS, 14 DSS) and harvested for ion analysis. Values indicate average ± SD (*n* = 3).

**FIGURE 6:**
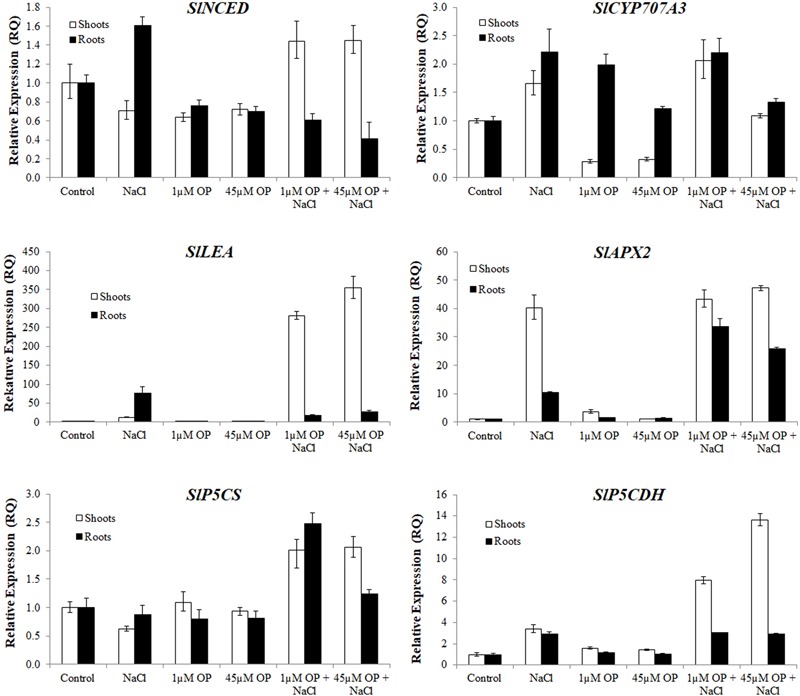
Secondary metabolism and stress signaling gene expression in plants treated with omeprazole. Plants were grown in a hydroponic solution containing 0, 1, and 45 μM OP, with and without 200 mM NaCl. Samples for qRT-PCR were harvested after 2 weeks of salt treatment (50 DAS, 14 DSS) and harvested for ion analysis. Values indicate average ± SD (*n* = 3).

**FIGURE 7:**
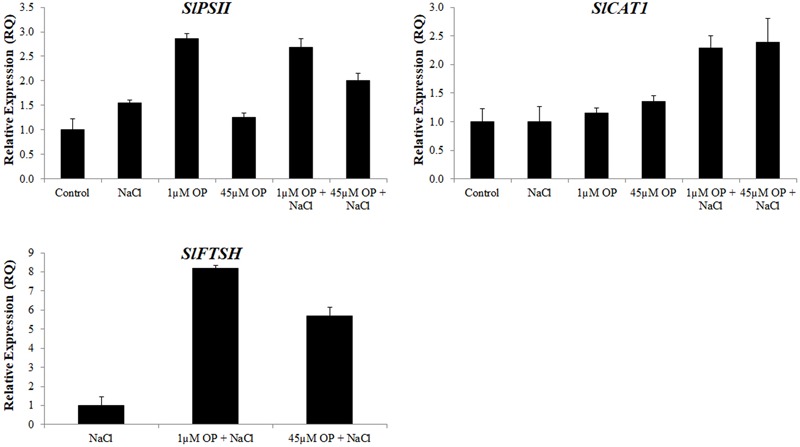
Photosynthetic gene expression in plants treated with omeprazole. Plants were grown in a hydroponic solution containing 0, 1, and 45 μM OP, with and without 200 mM NaCl. Samples from leaves for qRT-PCR were harvested after 2 weeks of salt treatment (50 DAS, 14 DSS) and harvested for ion analysis. Values indicate average ± SD (*n* = 3).

Gene expression analysis was used to characterize the downstream mechanisms affected by OP that resulted in improved growth and salt tolerance in hydroponically grown tomatoes (**Figures 5–7**). We looked at three general categories of genes: ion transporters, stress signal transduction and osmotic response components, genes involved in antioxidant and photosynthetic systems. Gene expression was evaluated in roots and shoots. We found that OP treatment affected a number of genes in non-stress conditions and augmented responses of numerous key genes involved in salinity stress responses and adaptation.

For ion accumulation, exclusions and transport, we selected a few ion transporters known to play key roles in responses to salinity: the plasma membrane Na^+^ antiporter SlSOS1, two tonoplast located K^+^ antiporters, SlNHX1 and SlNHX2 and the Na^+^ transporter SlHKT1.1 (**Figure 5**). SlSOS1 was significantly upregulated in all OP treatments, with augmented expression over untreated controls under salt stress in roots and shoots (**Figure 5**). This may have likely contributed to the lower Na^+^:K^+^ ratio seen in the ion analysis. For the two tonoplast located potassium antiporters, SlNHX1 and SlNHX2, which also mediate critical functions, including turgor maintenance, stomatal function and ion homeostasis under hyperosmotic stress (Zhang and Blumwald, 2001; Pardo et al., 2006), both genes were significantly upregulated under 1 μM OP treatment with increased expression over controls under salt stress (**Figure 5**). We also analyzed the expression pattern of HKT transporters, which play an important role in limiting the influx and subsequent accumulation of sodium into the shoot as well as sodium loading into root xylem (Asins et al., 2013; Ali et al., 2016). HKT1.1 expression in shoots was higher in OP treated plants compared to untreated controls in non-stress and salinity stress conditions (**Figure 5**). In roots, we found that OP treatment decreased SlHKT1.1 expression slightly in non-stress conditions and remarkably under salt stress. Decreased expression of HKT1 transporters may have significantly reduced sodium entry into roots, protecting them from ionic stress. Furthermore, HKT1 expression increased in salt stressed shoots. This may have favored sodium recirculation into the xylem, a function that coupled with decreased uptake and loading in roots could be a key role OP plays in salt tolerance. This result is consistent with a reduced Na^+^:K^+^ ratio found upon OP treatment. In non-stress conditions, treatment with OP increased the nitrate content of roots and moderately in shoots (**Figure 4**). In order to link the observed nitrate accumulation profile with gene functions, we examined the gene expression of the bidirectional transporter SlNRT1.1 responsible for uptake and transport of nitrate (Jossier et al., 2010). Expression of SlNRT1.1 was upregulated in OP treated plants, in roots and shoots, under non-stress conditions. We also observed higher SlNRT1.1 expression in salt stressed plants with OP application although no significant increases in nitrate content were detected under stress conditions.

With respect to stress signal transduction components, we analyzed the expression of an ABA biosynthesis gene, SlNCED, and an ABA catabolism gene SlCYP707A3. We found that OP treatment decreased SlNCED expression in roots and shoots in non-stress conditions. Interestingly, SlNCED was induced upon salt stress, yet OP treatment caused an opposite response compared to what was observed in control plants (**Figure 6**). Specifically, in contrast to control plants, shoot expression of SlNCED was elevated over untreated controls in shoots under salt stress while root expression was significantly reduced. SlCYP707A3 expression was highly dysregulated under 1 μM OP treatment. While shoot expression was less than a third of controls, root expression was nearly three times that of untreated roots. Under salinity stress and OP treatment, expression of SlCYP707A3 was not downregulated. To better explain the expression pattern of ABA related genes, we examined the expression of SlLEA a highly inducible marker in response to abiotic stress (Iovieno et al., 2016). While SlLEA was induced in salt stress conditions, it was less highly upregulated in the roots of OP treated plants under salt stress. This seems to correlate with decreased ABA signal transduction in the roots. In salt stressed shoots treated with OP, SlLEA demonstrated drastic upregulation, 10-fold higher than in untreated salt stress controls.

We also examined the antioxidant machinery and osmotic adaptation expression profiles of genes associated to ascorbate and proline biosynthesis. The cytosolic ascorbate peroxidase, SlAPX2, was highly upregulated in shoots and roots of salt stressed plants. In OP treated plants under salt stress, root SlAPX2 expression was highly induced, compared to salt stress controls, indicating a more robust ROS scavenging response induced by OP. With respect to proline and osmotic stress response, while expression of *pyrroline-5-carboxylate synthetase*, SlP5CS was not significantly altered in OP treated controls, it showed increased expression in salt stressed roots and shoots. Expression of the genes encoding for the catabolic pyrroline-5-carboxylate dehydrogenase, SlP5CDH, showed a similar pattern in shoots of OP treated plants under salt stress. This may have contributed to a differential accumulation of proline in the roots and shoots.

The last set of genes we examined were those involved in the protection of the photosynthetic system. Expression levels of the tomato photosystem II reaction center psb28-like protein (PSII) were significantly upregulated under low concentrations of OP. These increases were also observed in OP treated plants under severe salt stress (**Figure 7**). In the leaves of OP treated plants under salt stress we found significant upregulation of the tomato catalase gene, SlCAT1 (**Figure 7**). High SlCAT1 expression levels have been found to enhance salt stress tolerance by reducing photoinhibition from damage to the photosystem by H_2_O_2_ (Al-Taweel et al., 2007). We also examined the tomato homolog of FtsH, an ATP-dependent protease that plays a key role in degradation and repair of photosystem II (Kato et al., 2009; Sun et al., 2010). Expression of SlFTSH was below detectable thresholds in controls and OP treated plants. However, salt stress induced SlFTSH expression with an even greater upregulation under salt stress and OP treatment.

## Discussion

### Omeprazole Improves Plant Growth and Salt Stress Tolerance

In this work we demonstrated that by feeding tomato roots with hormonal concentrations of omeprazole, a benzimidazole PPI in animal systems, we can significantly improve plant growth and ability to tolerate saline stress. OP treatment with 1 μM increased shoot FW by 49% and DW by 48%. FW of roots was increased by 55% and DW by 56% in the absence of stress. Under saline stress, shoot growth was maintained, with a 56% increase in shoot FW and 54% increase in DW. Roots showed the most dramatic phenotype under salt stress, with a doubling of FW and DW over untreated controls (**Figures 1, 2**). Although this morphological change was not the only component that may have enhanced salt tolerance of OP treated plants, this response may have important implications with respect to growth and adaptation in saline environments (Julkowska et al., 2014; Feng et al., 2016). Longer, more extensive roots may help to escape salinization by exploring non salinized areas of the soil profile (De Pascale et al., 2012; Lynch et al., 2014; Feng et al., 2016; Annunziata et al., 2017). OP seems also to interfere with ABA responses. Lateral root formation is highly sensitive to ABA concentrations, with inhibition of lateral root primordia being an order of magnitude more sensitive than seed germination (De Smet et al., 2003). While ABA deficient mutants have impaired growth, endogenous ABA levels have been clearly shown to be inhibitory to root growth at low osmotic potentials (Sharp et al., 2004; Duan et al., 2013; Zhao et al., 2014). The effects of OP on lateral root formation are likely due to changes in ABA biosynthesis, catabolism, and/or perception. By decreasing biosynthesis of ABA in roots under salt stress and increasing catabolism, OP may overcome its inhibitory effects on root growth under low osmotic potential. These results also shed some light on the role of root systems in plant salt stress adaptation.

### Omeprazole Has Multiple Effects on Cellular Mechanisms That Enhance Salt Stress Tolerance

Hyperaccumulation of Na^+^ in the cytoplasm during salinity stress results in toxicity and disturbs essential cellular metabolisms such as protein synthesis, enzyme activity, and photosynthesis (Maggio et al., 2006; Hasegawa, 2013). Glycophytes cope with salinity stress by maintaining low cytosolic Na^+^ levels and by acquisition and maintenance of K^+^ (Flowers and Colmer, 2015). Sodium exclusion and potassium uptake are essential adaptations in response to high salinity in the environment that improve salt tolerance. OP appears to augment these adaptive mechanisms by affecting the regulation of a number of ion transporters. Under OP, increased expression of SlSOS1, SlNHX1, and SlNHX2 (**Figure 5**) establishes a pattern of sodium exclusion and increased potassium uptake, a result that was confirmed by the ion analysis (**Figure 3**). The plasma membrane sodium antiporter SOS1 is essential for excluding sodium from the cytoplasm and a key component in maintaining ion homeostasis (Ji et al., 2013). Similarly, NHX1 and NHX2 have been shown to enable maintenance of turgor, ion homeostasis, stomatal movements, growth regulation, cell expansion, and potassium uptake (Bassil et al., 2011; Barragán et al., 2012). In Arabidopsis, NXH1 selectivity has been associated to vacuolar calcium concentrations (Yamaguchi et al., 2005). The low concentrations of calcium found in shoot of OP treated plants at 1 and 10 μM may have likely been correlated to a reduced calcium entry into the roots and consequently to effects on the selectivity of NHX1, as confirmed by the low Na^+^:K^+^ ratio of root and shoot of OP treated plants (**Figure 3**). The expression pattern of SlHKT1.1 we found in root and shoot of OP treated plants is also of particular relevance. The dysregulation of SlHKT1.1 under salt stress and OP, with (1) increased expression in shoots to facilitate sodium recirculation to the roots and (2) decreased expression in roots to reduce sodium loading into the xylem and subsequent transport to sensitive photosynthetic tissues indicates that OP treatment augments the plant’s ability to control sodium accumulation in sensitive tissues (**Figure 5**). HKT transporters play an important role in limiting the influx of sodium into the shoot and subsequent accumulation as well as sodium loading into root xylem (Hauser and Horie, 2010; Ali et al., 2016). High sodium shoot accumulation has been linked to low AtHKT1.1 expression in roots in a number of Arabidopsis ecotypes (Rus et al., 2006). HKT1 transporters and non-selective cation channels (NSCCs) are the major contributors to sodium uptake in cells (Hasegawa, 2013). Decreasing the expression of HKT1 transporters in roots, while increasing HKT1 expression in shoots, could therefore be a key consequence of OP activity in plants under salt stress. Based on the ion profiles, it is clear that the phenotype of salt stress tolerance seen under OP treatment is due in part to a re-partitioning of ions under stress conditions. The increased expression of SlNRT1.1 (**Figure 5**) and the increased nitrate content in roots (**Figure 3**) indicate that OP may contribute to nitrogen uptake efficiency and resultant improvement in plant nutritional status. OP treated plants have a growth phenotype (**Figures 1, 2**) and increased nitrogen uptake would certainly contribute to increased growth in ideal conditions.

### OP Protects the Photosynthetic System

The reduction of A, g_s_ Φ_PSII_ and qP in non OP and OP treated plants exposed to 150 mM of NaCl compared to the 0 NaCl treatments indicated that salt stress reduced the efficiency of PSII reaction centers and impaired electron transport in the photosynthetic apparatus (Maxwell and Johnson, 2000; Baker, 2008). OP treatment seemed to improve the actual quantum yield of PSII (Φ_PSII_) and the photochemical quenching (qP) in the salt stressed leaves. We found that a number of key photosynthesis genes involved in photosystem II repair and ROS scavenging were upregulated under OP treatment (**Figures 6, 7**). Expression of catalase is a clear indicator of increased ROS scavenging and removal of potentially harmful accumulation of H_2_O_2_ (Mittova et al., 2000; Das and Roychoudhury, 2014). The upregulation under OP treatment of two key components of photosystem II repair, SlPSII and SlFTSH, seems to indicate that while salt stress does damage the photosystem, repair mechanisms required to maintain a nominal level of photosynthesis are less impeded. Transcript and protein accumulation of low molecular mass proteins (PSII like) have been observed in response to ROS and abiotic stress (Hihara et al., 2003; Kosmala et al., 2009; Shi et al., 2012). Arabidopsis mutants of SlPSII and SlFTSH genes show a decreased capacity for photosynthesis under abiotic stress (Sun et al., 2007) and in tomato, SlFTSH content is decreased after drought stress (Tamburino et al., 2017). The decreased expression of a key rate-limiting step of ABA biosynthesis in OP roots and shoots indicates that ABA levels are likely altered under OP treatment. More importantly, roots and shoots respond very differently under OP treatment, in the presence of salt stress. ABA responses under OP and salt treatment appear to be upregulated in shoots while at the same time, downregulated in roots. This is observed in the expression of SlNCED, SlCYP707A3, and the ABA responsive SlLEA gene. One possible explanation for this gene expression profile and observed growth phenotype under salt stress is that OP inhibited root ABA biosynthesis and activated shoot ABA biosynthesis which would allow root growth and branching under stress conditions (normally inhibited by ABA) (Sharp and LeNoble, 2002) and control ethylene production in the shoot which would otherwise inhibit growth (Sharp, 2002). This hypothesis could also be aligned with the expression levels of SlP5CS and SlP5CDH genes that may have contributed to an increased accumulation of proline in the roots, a response that typically, but not always, follows high ABA levels (Mattioli et al., 2009; Barbieri et al., 2012).

### Possible Targets of Omeprazole in Plants

At the moment we do not have yet conclusive evidence for the molecular target(s) of OP. The OP concentrations we used and dose responses indicate that OP acts with a hormone-like behavior with growth stimulation between 1 and 10 μM and inhibitory effects at higher concentrations (**Figures 1, 2**). Similar responses have been reported for other molecules including phytohormones (Bartoli et al., 2013; Huot et al., 2014; Lozano-Durán and Zipfel, 2015). However, only for a few of these single-molecule effectors a function on growth enhancement and stress tolerance has been demonstrated. Plants lack Type-IIC ATPases that transport K^+^ and Na^+^, the known target of OP, and the closest related classes of ATPases in plants share very low homology with animal H^+^/K^+^-ATPase (Sweadner and Donnet, 2001). However, based on the well-characterized function as H^+^/K^+^-ATPase inhibitor in animal systems, we can also hypothesize that OP is inhibiting an ATPase present in plants. This hypothesis is difficult to come to terms with, since very little room exists in our current paradigm of ATPase driven proton gradients and ion transport, where inhibition of one or more of these components would actually increase growth or tolerance to salinity. Ion homeostasis is key to growth and adaptation to osmotic stress, a clear mechanism for the role of OP does not readily present itself. The possibility that OP is exerting its effect through a mechanism of action which is unrelated to an ATPase inhibitory function in plants should also be considered. OP appears to be one of a few molecules with a dual function of growth enhancer and stress protectant and it represents an excellent candidate to explore key mechanisms that could shed some light on how plant growth inhibition and adaptation in response to salt stress can be uncoupled (Ruggiero et al., 2004). While the exact target of omeprazole remains unclear, the physiological effects open new avenues for understanding the mechanisms that allow plants to grow under adverse conditions.

## Author Contributions

MVO wrote the paper and did most of the experimental work. SS contributed to plant growth analyses and molecular characterization. GG made the gas exchange measurements and fluorescence analysis. VC and EDS worked on the statistical analysis. PC and PW worked on ion analysis and contributed to writing. AM wrote the paper together with MVO and coordinated the research work. GR had the original idea on testing omeprazole on plants and contributed to data analysis.

## Conflict of Interest Statement

The authors declare that the research was conducted in the absence of any commercial or financial relationships that could be construed as a potential conflict of interest.

## Footnotes

1 http://tgrc.ucdavis.edu/

2 http://www.ncbi.nlm.nih.gov/genbank
