# Supplementary material for: A Benzimidazole Proton Pump Inhibitor Increases Growth and Tolerance to Salt Stress in Tomato
Source: Front Plant Sci. 2017 Jul 18;8:1220. doi: 10.3389/fpls.2017.01220 (PMC5513968; doi:10.3389/fpls.2017.01220)

**A benzimidazole proton pump inhibitor increases growth and tolerance to salt stress in tomato**

AUTHORS: Michael James Van Oosten^1^, Silvia Silletti^1^, Gianpiero Guida^2^, Valerio Cirillo^1^, Emilio Di Stasio^1^, Petronia Carillo^3^, Pasqualina Woodrow^3^, Albino Maggio^1*^, Giampaolo Raimondi^1^

^1^ Department of Agriculture Sciences, University of Naples Federico II, Portici (NA), Italy

^2^ National Research Council of Italy, Institute for Agricultural and Forestry Systems in the Mediterranean (CNR-ISAFoM), Ercolano, Italy

^3^Department of Environmental, Biological and Pharmaceutical Sciences and Technologies of University of Campania “Luigi Vanvitelli”, Caserta, Italy

Table S1: List of primers used in this study.

| Primer Name | Sequence | | Accession # | |
| --- | --- | --- | --- | --- |
| Sl707A1-F | CTGAACAGAAAGTTATTTGGCAGTC | | [Solyc04g078900.2](https://solgenomics.net/tools/blast/show_match_seq.pl?blast_db_id=224;id=Solyc04g078900.2;hilite_coords=61-1754) | |
| Sl707A1-R | ATGATACTAGCCATTCTCAGTGTCTC | | [Solyc04g078900.2](https://solgenomics.net/tools/blast/show_match_seq.pl?blast_db_id=224;id=Solyc04g078900.2;hilite_coords=61-1754) | |
| Sl707A3-F | GCTCCCAAACCCAATACCTAC | | [Solyc04g071150.2](https://solgenomics.net/tools/blast/show_match_seq.pl?blast_db_id=224;id=Solyc04g071150.2;hilite_coords=1595-3379) | |
| Sl707A3-R | CAGTTTGGCGAGTTCATTTCC | | [Solyc04g071150.2](https://solgenomics.net/tools/blast/show_match_seq.pl?blast_db_id=224;id=Solyc04g071150.2;hilite_coords=1595-3379) | |
| SlHKT1.1-F | TCTAGCCCAAGAAACTCAAAT | | [Solyc07g014680.2](https://solgenomics.net/tools/blast/show_match_seq.pl?blast_db_id=224;id=Solyc07g014680.2;hilite_coords=33-677) | |
| SlHKT1.1-R | CTAATGTTACAACTCCAAGGAATT | | [Solyc07g014680.2](https://solgenomics.net/tools/blast/show_match_seq.pl?blast_db_id=224;id=Solyc07g014680.2;hilite_coords=33-677) | |
| SlNCED1-F | CATAATCGAAAACCCGGATG | | Solyc07g056570.1 | |
| SlNCED1-R | AACTTTTGGCCATGGTTCAG | | Solyc07g056570.1 | |
| SlNHX1-F | CACGATATGGTGGGCTGGTT | | Solyc06g008820.2 | |
| SlNHX1-F | GGGTGTGGCCAAATCTCGTA | | Solyc06g008820.2 | |
| SlNHX2-F | ATTGGAGGATCGGCAGGAAC | | Solyc04g056600.2 | |
| SlNHX2-R | CCATGGAGCCAGATTGACCA | | Solyc04g056600.2 | |
| SlNRT1.1-F | TAGCGCCGCGATGATATTAGGG | | Solyc06g074990 | |
| SlNRT1.1-R | TGTAACGTTGTTGGCTGAACTTGC | | Solyc06g074990 | |
| SlP5CD-F | CACAGGTAGCTCAAGGGTGG | | Solyc02g089620 | |
| SlP5CD-R | GGCCCAAGGATCTTCCAGTC | | Solyc02g089620 | |
| SlP5CS-F | AACTGAGCTTGATGGCAAGG | | Solyc08g043170 | |
| SlP5CS-R | ACCAGAGGCTGAGCTGATGT | | Solyc08g043170 | |
| SlSOS1-F | TCGAGTGATGATTCTGGTGG | | Solyc01g005020 | |
| SlSOS1-R | ATCACAGTGTGGAAAGGCT | | Solyc01g005020 | |
| SlSLEA-F | CGAAGGAGAAGGCTAGTGGA | | Solyc03g116390.2 | |
| SlSLEA-R | AGCGATGCTCCTCACTTGTT | | Solyc03g116390.2 | |
| SlAPX2-F | ATGGTAGCTGGAGGAGACCT | | Solyc06g005150 | |
| SlAPX2-R | TTGAGGGAGCATGGACCAAC | | Solyc06g005150 | |
| SlCAT1-F | GGACAATAATGGCAGGGCAA | | Solyc12g094620 | |
| SlCAT1-R | TTACAGCCAGTTGGTCGCTT | | Solyc12g094620 | |
| SlCHLBP-F | GAGCATTCTAGCAGTATTGG | | Solyc07g063600 | |
| SlCHLBP-R | TGTTGCCTTCACCAACTCCA | | Solyc07g063600 | |
| SlPSII-F | CGAAGAAGGGGTTCTAACGT | | Solyc09g064500 | |
| SlPSII-R | GTCTCGTGGAGTCCGCATAA | | Solyc09g064500 | |
| SlPFTSH-F | AAGGACGTGGATCTGTCAGC | | Solyc03g112590 | |
| SlPFTSH-R | TGGCATATTTGCATGCTCGC | | Solyc03g112590 | |
|  |  |  | |  |

Figure S1: Photosynthetic rates (A) and stomatal conductance (g_s_) of OP treated and control plants. Plants were grown in soil, unsalinized or salinized with 150 mM NaCl and irrigated with 0 and 1 µM OP. Photosynthetic rates (top panels) and stomatal conductance (bottom panels) were taken before and after salt stress. Values indicate average ± SE (n=6).


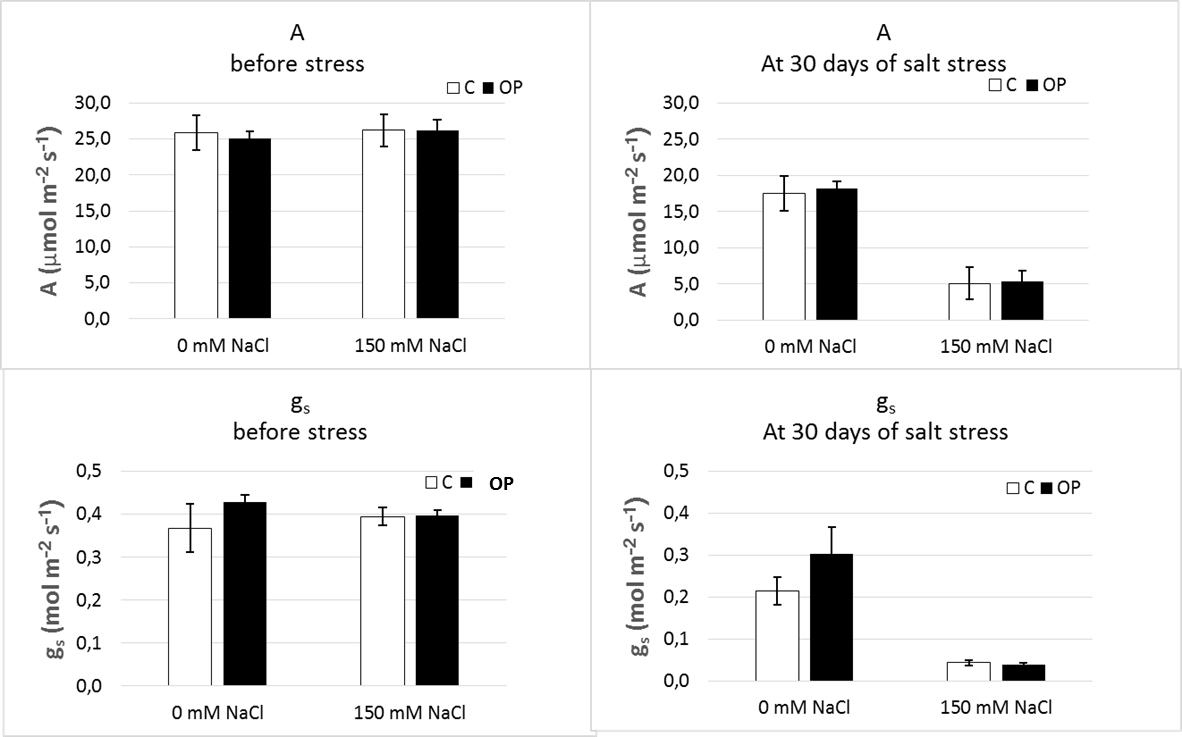

Supplement: Supplementary file 1 [file Table_1.DOCX]
